# Oral administration of aripiprazole to *Drosophila* causes intestinal toxicity

**DOI:** 10.1242/dmm.052180

**Published:** 2025-03-24

**Authors:** James D. Hurcomb, Amrita Mukherjee, Anna E. Lindell, Rebeka Popovic, Yizhou Yu, Kiran R. Patil, Samantha H. Y. Loh, L. Miguel Martins

**Affiliations:** MRC Toxicology Unit, University of Cambridge, Gleeson Building, Tennis Court Road, Cambridge CB2 1QR, UK

**Keywords:** *Drosophila*, Intestine, Mitochondria, JNK signalling, Reactive oxygen species, Antipsychotics, Aripiprazole, Mitochondrial respiratory complex I

## Abstract

Aripiprazole is a third-generation antipsychotic medication that was introduced to mitigate the poor tolerability of older antipsychotics. In contrast to the older antipsychotic drugs that act as dopamine receptor antagonists in the brain, aripiprazole functions as a partial agonist. Aripiprazole has been identified as an off-target inhibitor of mitochondrial respiratory complex I. We observed that patients prescribed aripiprazole often report gastrointestinal disturbances, but the mechanism underlying these side effects is not clear. We modelled the potential mitochondrial toxicity of aripiprazole in the gastrointestinal system using the fruit fly (*Drosophila melanogaster*). Aripiprazole consumption impaired *Drosophila* gut function and faecal output. It also reduced the mitochondrial membrane potential and increased reactive oxygen species (ROS) levels in intestinal cells. ROS activate the c-Jun N-terminal kinase (JNK) pathway, which induces cellular stress and cell death. Aripiprazole increased JNK activation in the intestinal cells of flies, resulting in cell death, which was suppressed by antioxidants. We conclude that aripiprazole activates the JNK pathway of cell death via mitochondrial ROS production. Using antioxidant supplements may help reduce aripiprazole-induced toxicity.

## Teaser

**Summary:** Aripiprazole-induced gut toxicity in *Drosophila melanogaster* is mediated by mitochondrial complex I inhibition and JNK-dependent apoptosis.

## INTRODUCTION

Aripiprazole functions as a partial agonist of the dopamine D2 receptor (Shapiro et al., 2003). Partial agonism stabilises dopaminergic signalling, unlike the mechanism of action of earlier antipsychotics. This mechanism is expected to enhance efficacy and minimise side effects (Bhattacharjee and El-Sayeh, 2008).

Despite its advantages, aripiprazole still has significant side effects. We found that aripiprazole inhibits mitochondrial respiratory complex I, which was a previously unknown off-target effect (Hardy et al., 2023). Gastrointestinal distress is a common side effect of aripiprazole, prompting our investigation into a potential connection with mitochondrial toxicity from this medication. We modelled the gastrointestinal side effects of aripiprazole in *Drosophila* and assessed the potential role of mitochondrial toxicity in its reported side effects.

*Drosophila* can be used as a model organism for studying gut toxicity caused by orally administered drugs. *Drosophila* share conserved cytochrome P450 enzymes (CYP450s) with humans, which are critical for drug metabolism. Structural modelling and docking comparisons show significant similarities between fruit fly and human CYP450s, which underscore the relevance of the fruit fly for studying drug metabolism and toxicity (Nirusimhan et al., 2022). *Drosophila* are genetically tractable, which allows for rapid and cost-effective genetic manipulation to study the effects of specific genes on drug toxicity. This advantage is significant for identifying the genetic basis of drug responses and toxicity (Pandey and Nichols, 2011).

The intestinal system of *Drosophila* is divided into three main regions: the foregut, the midgut and the hindgut. These correspond to the oesophagus, the small intestine and the large intestine of mammals, respectively. The intestinal system of *Drosophila* is composed of three main cell types: enterocytes, intestinal stem cells and enteroendocrine cells (reviewed in Miguel-Aliaga et al., 2018). Reactive oxygen species (ROS) are chemically reactive molecules that are generated as natural byproducts of the normal metabolism of oxygen. ROS play a critical role in cell signalling and homeostasis (reviewed in Ray et al., 2012). Excessive ROS levels can lead to oxidative stress that damages cellular components and activates various stress response pathways. ROS stimulate a highly conserved signalling cascade that involves the phosphorylation and activation of c-Jun N-terminal kinase (JNK), which translocates to the nucleus to influence gene expression. The JNK pathway in *Drosophila*, which specifically involves the JNK orthologue encoded by the gene *basket* (*bsk*), is a well-conserved mechanism that is activated by oxidative stress. The activation of Bsk leads to the transcriptional upregulation of the gene *puckered* (*puc*), which encodes a dual-specificity phosphatase that dephosphorylates and inactivates Bsk to create a negative feedback loop that regulates the intensity and duration of JNK signalling (Martin-Blanco et al., 1998). The JNK signalling pathway regulates tissue homeostasis in the *Drosophila* intestine, and its chronic activation causes apoptotic cell death and tissue degeneration (Mundorf et al., 2019).

Here, we explored the potential toxicity of aripiprazole to the intestinal system. By querying the US Food and Drug Administration (FDA) Adverse Event Reporting System (FAERS) database, we found that many patients taking aripiprazole reported gastrointestinal issues. Earlier studies showed that mice kept on an aripiprazole-supplemented diet exhibited morphological changes in the small intestine (Baek et al., 2015). Using the fruit fly, we show that the oral administration of aripiprazole causes intestinal toxicity. We found that this toxicity was caused by disruption of mitochondrial function, which increased the levels of ROS, JNK signalling and apoptosis in the *Drosophila* gut. We also found that increasing antioxidant defences in the gut blocked the aripiprazole-induced toxicity. We propose that the gastrointestinal side effects of aripiprazole could be attributed to intestinal dysfunction caused by ROS-dependent JNK activation.

## RESULTS

### Intestinal dysfunction in aripiprazole-supplemented flies

**Fig. 1.**
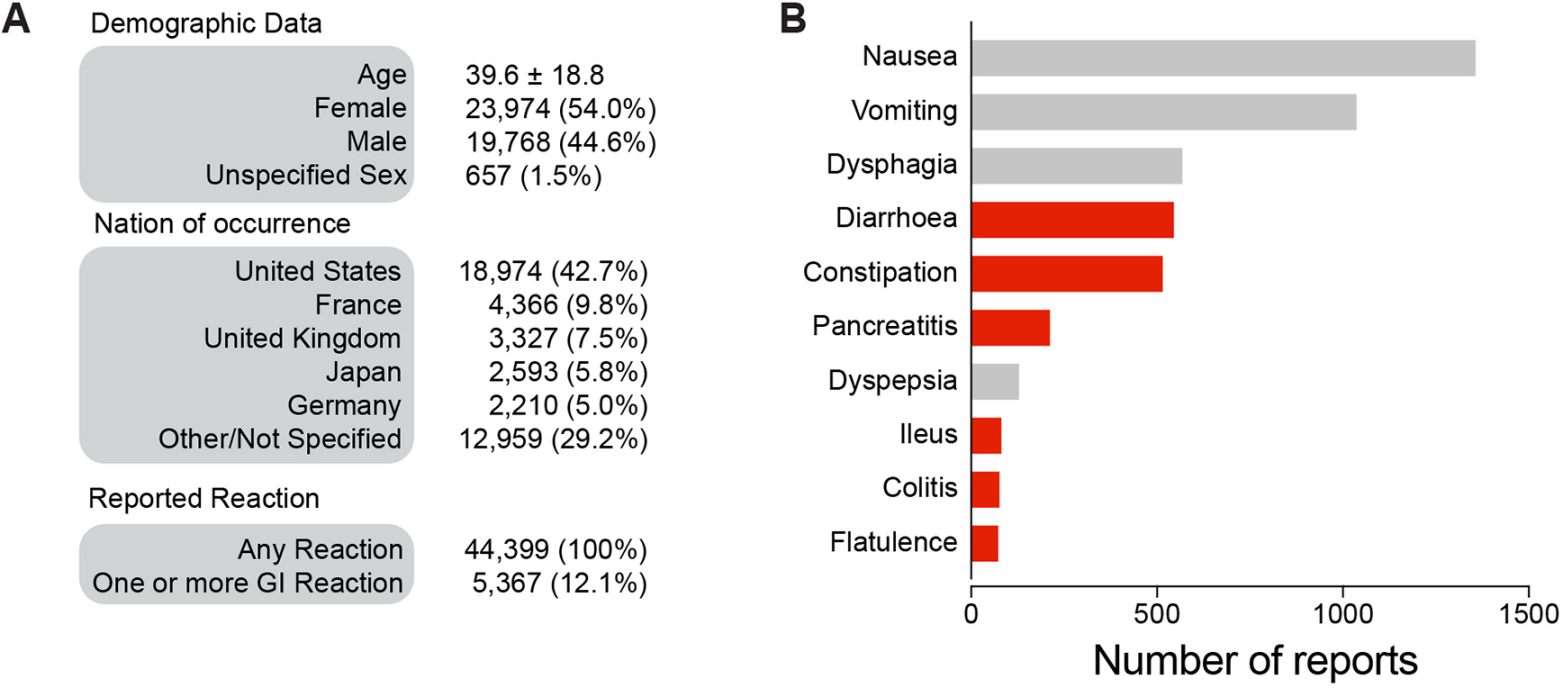
**Adverse events reported by patients prescribed aripiprazole to the FDA.** (A) Demographics and geographical location of patients who reported side effects of aripiprazole to the US Food and Drug Administration (FDA) (see also Materials and Methods). More than 10% of the reported side effects involved a gastrointestinal disturbance. (B) Six of the top ten most reported gastrointestinal side effects impacted the lower gastrointestinal (GI) tract (highlighted in red).

Aripiprazole is a third-generation antipsychotic drug that is regularly administered to patients with psychiatric conditions, often via an oral route (Sullivan et al., 2007). We recently reported that aripiprazole causes cellular toxicity by inhibiting mitochondrial function (Hardy et al., 2023). Assessing the potential for gut toxicity in orally administered aripiprazole is fundamental for safeguarding patient health, optimising treatment effectiveness and guiding pharmaceutical development and regulatory approval processes. These processes ensure that medications are safe and effective for consumer use. To analyse the self-reported side effects associated with aripiprazole intake, we first queried the FAERS database (see Materials and Methods) and found that approximately 10% of patients taking this drug reported gastrointestinal problems (Fig. 1).

**Fig. 2.**
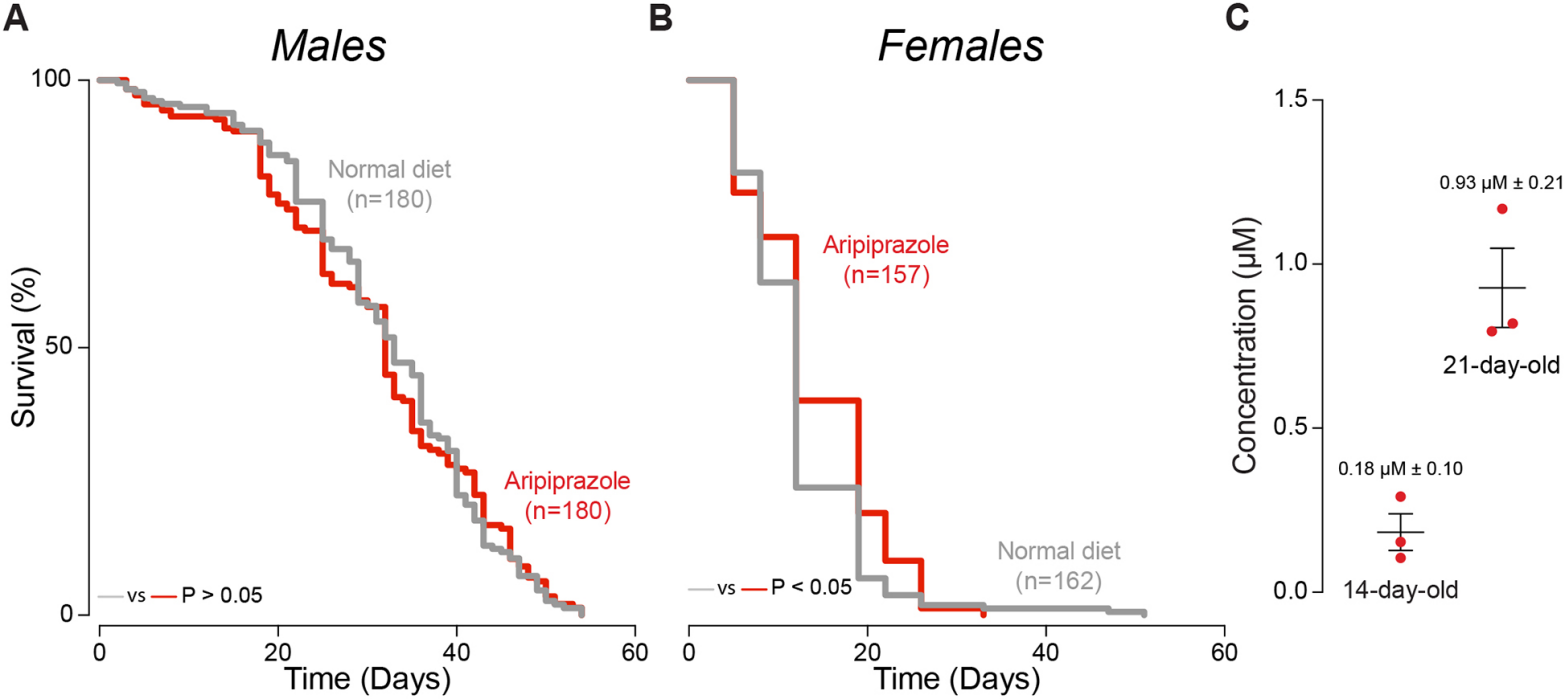
**Analysis of lifespan and aripiprazole levels in flies.** (A,B) An aripiprazole-containing diet did not alter the lifespan of male flies (A) but shortened the lifespan of female flies (B). Significance was determined using the log-rank (Mantel–Cox) test. (C) Aripiprazole was detected in male flies using mass spectrometry, and the concentration increased over the 21 days the flies were kept on an aripiprazole-supplemented diet. Bars show mean±s.d. Genotype: *w^1118^CS* (A-C).

*Drosophila* is an excellent model organism for studying gut toxicity (reviewed in Apidianakis and Rahme, 2011). We therefore used this insect model to determine the molecular and cellular consequences of the oral administration of aripiprazole. We kept flies on a diet supplemented with aripiprazole and measured their lifespan. The lifespan of male flies remained unchanged with aripiprazole supplementation, whereas an aripiprazole-supplemented diet reduced the lifespan of female flies (Fig. 2A,B).

In humans, aripiprazole is detected in the plasma at concentrations ranging from 150 to 210 ng/ml (0.33 to 0.46 µM), and it has moderate to severe side effects at concentrations between 210 and 335 ng/ml (0.46 to 0.74 µM) (Sparshatt et al., 2010). We next kept adult flies on an aripiprazole-supplemented diet and measured its concentration in whole-fly extracts using mass spectrometry (see Materials and Methods). We found that aripiprazole reached concentrations ranging from 0.10 µM in 14-day-old flies to 1.7 µM in 21-day-old flies (Fig. 2C).

**Fig. 3.**
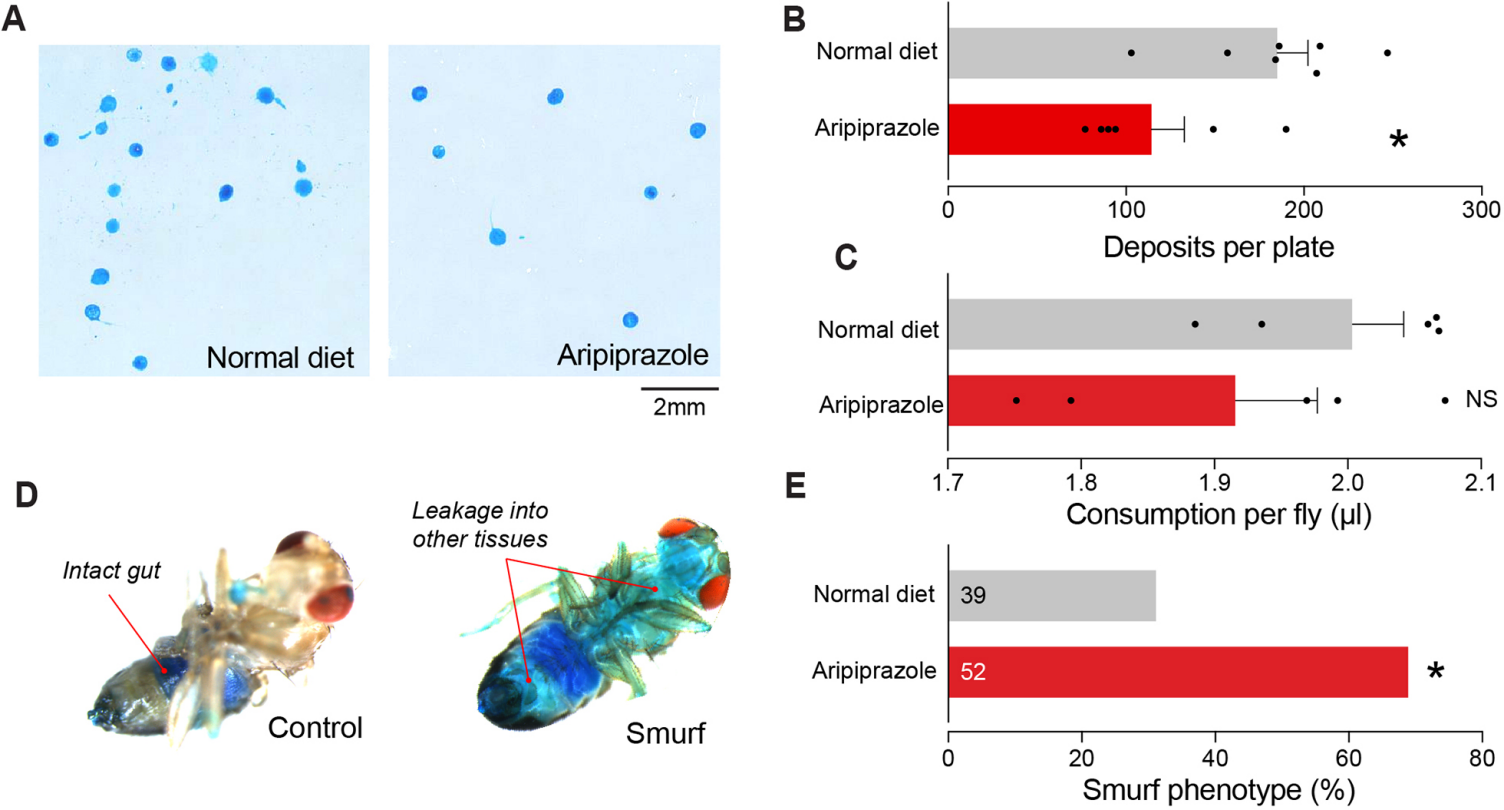
**Flies kept on a diet supplemented with aripiprazole show physiological markers of gut dysfunction.** (A,B) Aripiprazole altered the number of faecal deposits produced by *Drosophila*. (A) Representative images of fly faeces (over an 18-h period of consuming food supplemented with blue dye to aid in the visualisation of faeces). (B) Quantification of the number of faecal deposits per plate containing five flies. Bars show mean±s.e.m. *P≤0.05 (unpaired two-tailed *t*-test). (C) A diet supplemented with aripiprazole did not alter the food intake of adult flies. In this assay, flies were kept on an aripiprazole-supplemented diet for 14 days, and then provided with a liquid diet over an 18-h period. Bars show mean±s.e.m. NS, not significant; *P*>0.05 (unpaired two-tailed *t*-test). (D,E) Aripiprazole impaired the integrity of the intestinal barrier in flies. Representative images (D) showing the Smurf phenotype and quantification (E) of the percentage of flies showing the Smurf phenotype. *P≤0.05 (Fisher's exact test). Genotype: *w^1118^CS* (A-E).

We next determined whether chronic supplementation of flies with aripiprazole in their diet compromised intestinal function. We analysed both food intake and defecation rates using established methods (Cognigni et al., 2011; Ja et al., 2007). We found that aripiprazole-supplemented flies showed a reduction in the number of faecal deposits (Fig. 3A,B), with no significant changes in food intake (Fig. 3C). Gut dysfunction is often associated with a loss of the integrity of the intestinal barrier in flies. We next used the Smurf assay, which is a non-invasive method to determine the integrity of the intestine of adult flies. The Smurf assay showed that aripiprazole compromised the integrity of the intestinal barrier (Fig. 3D,E). Taken together, these data show that chronic supplementation of adult flies with aripiprazole results in body concentrations comparable to those in humans and compromises intestinal function without altering feeding.

### An aripiprazole-supplemented diet causes mitochondrial toxicity in intestinal cells

**Fig. 4.**
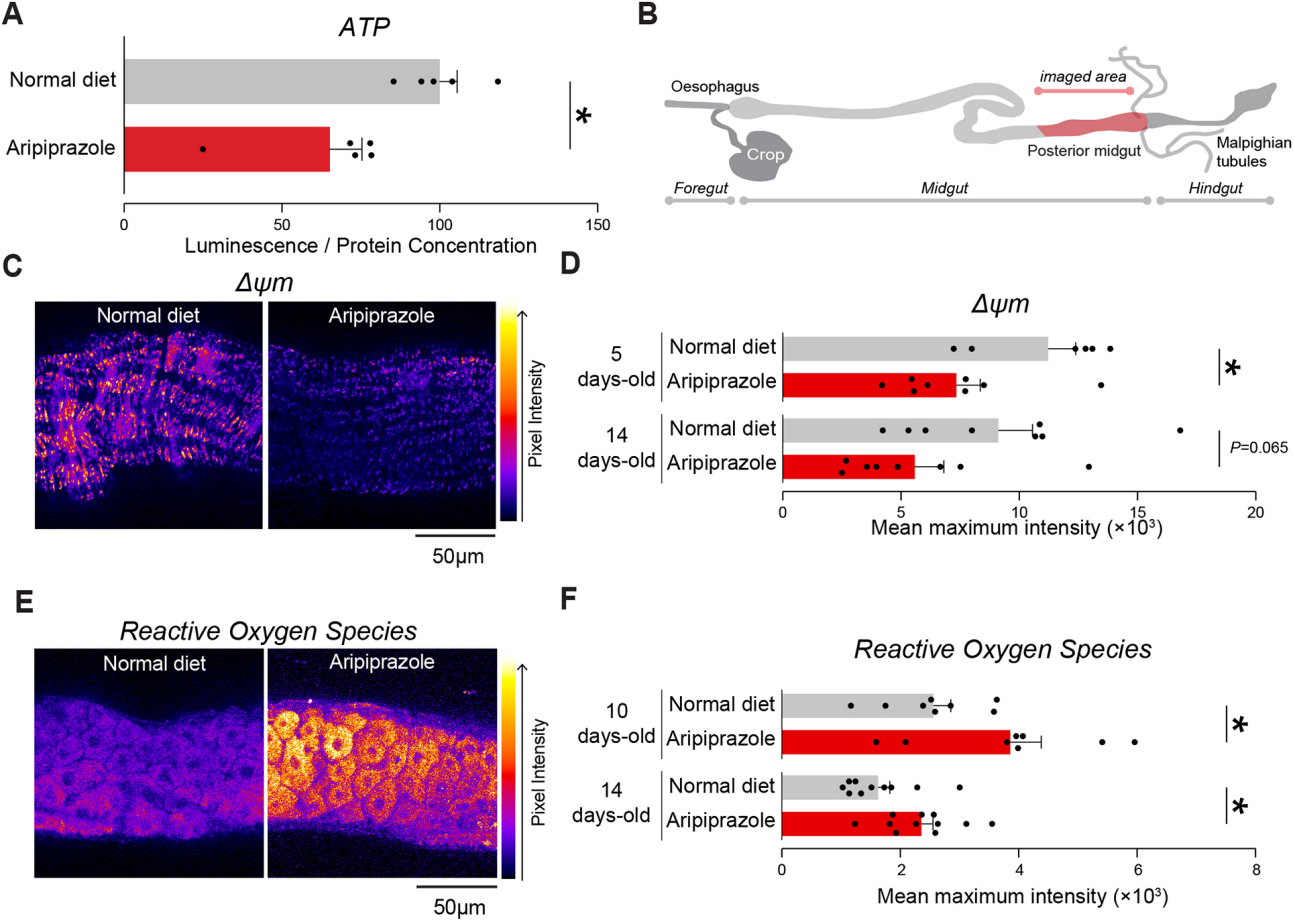
**Aripiprazole-supplemented flies show impaired mitochondrial function and increased ROS levels in intestinal cells.** (A) Lower levels of ATP were detected in flies kept on a diet supplemented with aripiprazole. Bars show mean±s.e.m. *P≤0.05 (unpaired two-tailed *t*-test). (B) Schematic of the adult *Drosophila* gut showing the region of interest that was imaged in C and E. (C,D) A lower mitochondrial membrane potential (Δψm) was observed in the intestine of aripiprazole-supplemented flies. (C) Representative images with tetramethylrhodamine methyl ester (TMRM) intensity levels mapped using the ‘Fire’ lookup table in FIJI. (D) Quantification of TMRM intensity levels in 5- and 14-day-old male flies. Bars show mean±s.e.m. *P≤0.05 (unpaired two-tailed *t*-test). (E,F) Increased levels of mitochondrial reactive oxygen species (ROS) were detected in the intestines of aripiprazole-supplemented flies. (E) Representative images with mitoSOX intensity levels mapped using the Fire lookup table in FIJI. (F) Quantification of mitoSOX intensity levels in male flies. Bars show mean±s.e.m. *P≤0.05 (unpaired two-tailed *t*-test). Genotype: *w^1118^CS* (A-F).

Aripiprazole acts as an off-target inhibitor of mitochondrial respiratory complex I, and flies kept on a diet supplemented with aripiprazole were found to have damaged mitochondria in their brain and muscle tissues (Hardy et al., 2023) and a reduction in their ATP levels (Fig. 4A). Therefore, we determined whether oral supplementation of aripiprazole compromised mitochondrial function in intestinal cells. The mitochondrial membrane potential (Δψm) is an indicator of mitochondrial health and function. We measured the Δψm of intestinal cells using tetramethylrhodamine methyl ester (TMRM). The midgut of flies kept on an aripiprazole-supplemented diet showed a decrease in the Δψm, which indicated that this drug impaired mitochondrial function in the intestine (Fig. 4C,D).

Complex I is the main site of ROS production in mitochondria (Kushnareva et al., 2002). Excessive levels of ROS produced by complex I can lead to apoptosis, which is a form of programmed cell death (Li et al., 2003). We next measured mitochondrial ROS levels in the guts of flies kept on a diet supplemented with aripiprazole and found a significant increase in their ROS levels (Fig. 4E,F).

We concluded that flies kept on a diet supplemented with aripiprazole compromised the mitochondria of intestinal cells and led to an increase in mitochondrial ROS.

### Ectopic expression of an alternative NADH dehydrogenase from yeast improves mitochondrial health in aripiprazole-supplemented flies

**Fig. 5.**
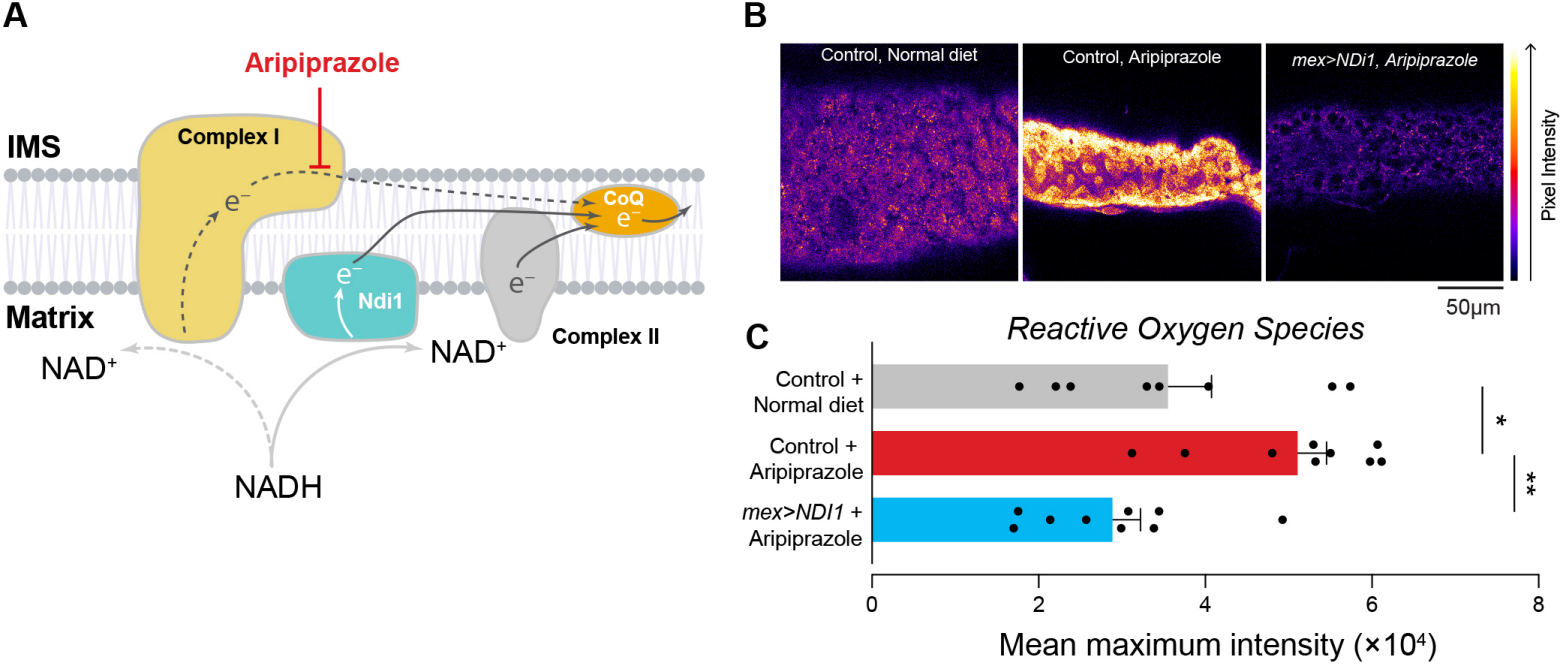
**Expression of the yeast alternative oxidase *NDI1* decreases ROS levels caused by aripiprazole.** (A) Yeast Ndi1 can bypass dysfunctional mitochondrial respiratory complex I in flies. Complex I receives electrons from the oxidation of NADH to NAD^+^. Aripiprazole acts at the binding site for ubiquinone (CoQ), preventing passage of electrons further through the respiratory chain (Hardy et al., 2023). Ndi1 is able to bypass impaired complex I, oxidising NADH and transferring electrons to CoQ and the downstream components of the respiratory chain. IMS, intermembrane space. (B,C) Reduced levels of mitochondrial ROS were detected in the intestines of flies expressing *NDI1* kept on an aripiprazole-containing diet. (B) Representative images with mitoSOX intensity levels mapped using the Fire lookup table in FIJI. (C) Quantification of mitoSOX intensity levelsin 14-day-old male flies. Bars show mean±s.e.m. *P≤0.05; **P≤0.01 (one-way ANOVA with Holm-Šídák's multiple comparisons test). Genotypes: *w; mex1-Gal4/UAS3M; +* (control), *w; mex-1Gal4; UAS-NDI1* (*mex>NDI1*).

Plants and fungi possess an alternative NADH dehydrogenase, Ndi1, on the matrix side of the mitochondrial inner membrane. It oxidises NADH and reduces ubiquinone, thus bypassing complex I (reviewed in Hernansanz-Agustin and Enríquez, 2023). Flies ectopically expressing *NDI1* are protected from mitochondrial toxicity caused by complex I inhibitors (Sanz et al., 2010). We observed that expressing *NDI1* in the enterocytes of flies kept on an aripiprazole-supplemented diet reduced the increase in ROS levels observed in the intestinal cells of control flies kept on an aripiprazole-containing diet (Fig. 5). We concluded that bypassing complex I inhibition in flies protects intestinal cells from the mitochondrial off-target toxicity of aripiprazole.

### Aripiprazole exposure activates the JNK pathway

**Fig. 6.**
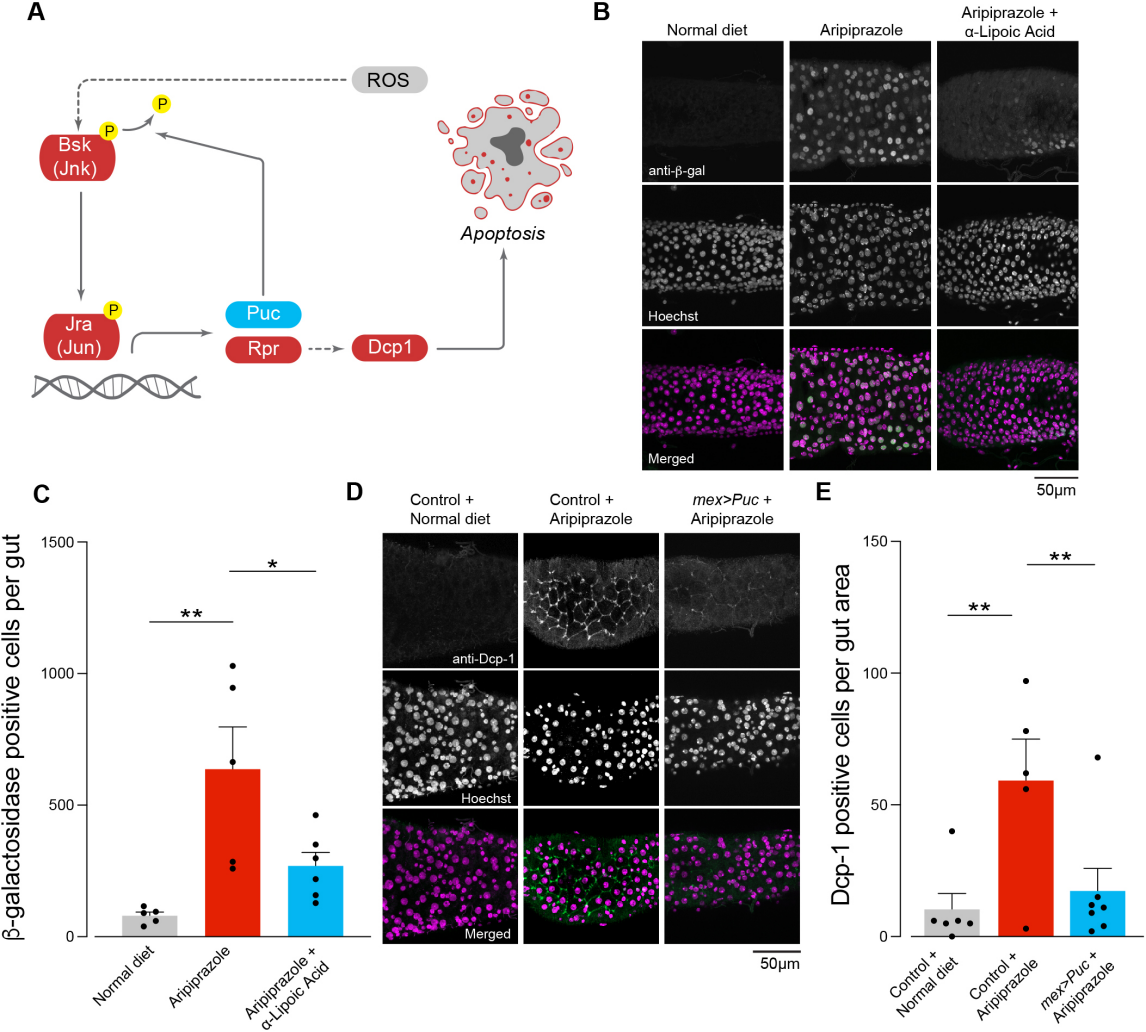
**Activation of the JNK signalling pathway in the intestine of flies kept on a diet supplemented with aripiprazole.** (A) Schematic of the JNK pathway in *Drosophila*. Stressors, including ROS, activate Basket (Bsk), a c-Jun N-terminal linked kinase (Jnk). The activation of Bsk causes widespread changes in transcription, including the transcription of the *puckered* gene encoding the phosphatase Puc, which acts as a negative regulator to prevent further pathway activation, and the *reaper* gene encoding Rpr, which activates apoptosis pathways. The dashed line indicates that the whole pathway is not shown. (B,C) Aripiprazole activates the JNK pathway in the intestine of drug-fed flies, and this activation was reduced by the supplementation of antioxidants. (B) Representative confocal images of the posterior midgut of *Drosophila* showing JNK activity visualised using an anti-β-galactosidase antibody (green) and cell nuclei (magenta). (C) Quantification of the number of β-galactosidase-positive nuclei in the entire midgut from the proventriculus region to the posterior midgut-hindgut junction. Bars show mean±s.e.m. *P≤0.05; **P≤0.01 (one-way ANOVA with Tukey's multiple comparisons test). (D,E) Ectopic expression of Puc (Martin-Blanco et al., 1998) rescues cell death in the posterior midgut of flies kept in an aripiprazole-containing diet. (D) Representative confocal images of the posterior midgut of *Drosophila* showing showing active Dcp-1 (green) and cell nuclei (magenta). (E) Quantification of active Dcp-1-positive cells in the posterior midgut. Bars show mean±s.e.m. **P≤0.01 (one-way ANOVA with Holm-Šídák's multiple comparisons test). Genotypes: *w; +; puc^E69^/TM3, Sb^1^* (B,C); *w; mex1-Gal4/UAS3M; +* (control) and *w; mex1-Gal4; UAS-Puc* (*mex>Puc*) (D,E).

The JNK pathway (Fig. 6A) is an evolutionarily conserved stress response pathway triggered by stressors, such as ROS. The *Drosophila* JNK pathway involves a single JNK gene, *bsk*, which, upon activation, leads to the transcription of target genes to reduce cellular stress or induce apoptosis. Activation of the JNK pathway also causes transcription of the gene *puc*, which negatively regulates this pathway (Fig. 6A) (reviewed in Tafesh-Edwards and Eleftherianos, 2020). The JNK pathway is essential for conferring protection against ROS in *Drosophila* (Wang et al., 2003). We therefore monitored JNK activity in the entire *Drosophila* midgut using a *puckered lacZ* enhancer trap line (Martin-Blanco et al., 1998) and measured the levels of β-galactosidase in gut cells by immunofluorescence. Aripiprazole-supplemented flies showed an increase in JNK pathway activity compared to control flies kept on a normal diet (Fig. 6B,C).

To test whether the increase in JNK activity was a result of an increase in ROS, we kept flies on a diet containing both aripiprazole and an antioxidant, α-lipoic acid. We found that the flies given a diet containing both α-lipoic acid and aripiprazole showed a decrease in the number of β-galactosidase-positive cells in the intestine compared to flies kept on a diet supplemented with aripiprazole alone (Fig. 6B,C). Thus, we concluded that the increase in ROS caused by aripiprazole activates the JNK pathway, which can be suppressed by an antioxidant-supplemented diet.

### Aripiprazole causes JNK-dependent intestinal cell death

In vertebrates, the JNK pathway regulates the intrinsic pathway of apoptosis. In *Drosophila*, activation of this pathway results in the transcriptional upregulation of pro-apoptotic genes, including *reaper*, *hid* and *grim* (reviewed in Dhanasekaran and Reddy, 2008). Reaper, Hid and Grim indirectly activate the initiator caspases, which in turn activate effector caspases, such as Dcp-1, to execute the apoptotic programme in *Drosophila*. We therefore monitored the levels of active Dcp-1 in the posterior midgut of aripiprazole-supplemented flies (Fig. 6D,E) and found increased levels of active Dcp-1. To confirm that the caspase activation was a consequence of JNK activation, we overexpressed *puc* in enterocytes to inhibit this pathway. The *puc* gene encodes Puckered (Puc), a phosphatase that dephosphorylates and inactivates Jnk (Fig. 6A). We found that in flies kept on an aripiprazole-containing diet, the overexpression of *puc* in enterocytes lowered the number of active Dcp-1-positive cells (Fig. 6D,E). We conclude that JNK activation is required for aripiprazole-dependent apoptosis of intestinal cells.

### An aripiprazole diet supplemented with antioxidants reduces intestinal cell death and restores gut integrity

**Fig. 7.**
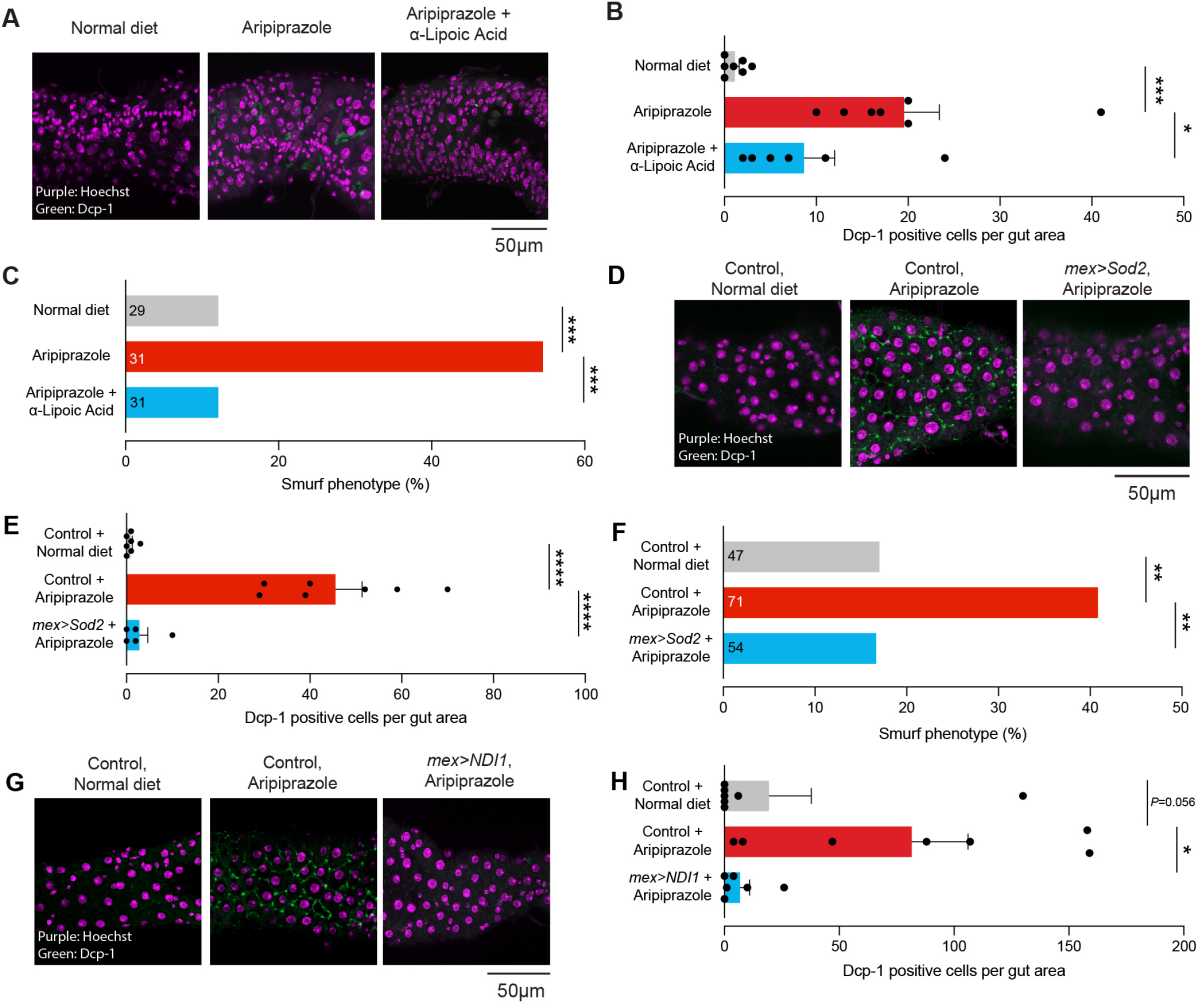
**Dietary antioxidant supplementation reverses intestinal dysfunction in aripiprazole-supplemented flies.** (A,B) An antioxidant-supplemented diet (α-lipoic acid) reduced the number of cells positive for the active caspase Dcp-1 in *Drosophila* kept on an aripiprazole-supplemented diet. (A) Representative images showing active Dcp-1 (green) and cell nuclei (magenta). (B) Quantification of active Dcp-1 in the posterior midgut. Bars show mean±s.e.m. *P≤0.05; ***P≤0.001 (one-way ANOVA with Holm-Šídák's multiple comparisons test). (C) Defects in the intestinal barrier observed in aripiprazole-supplemented flies were reduced by antioxidant consumption. ***P≤0.001 (χ^2^ two-sided test with analysis of a stack of *P*-values, FDR=10%; control versus aripiprazole, χ^2^=12.65, *P*=0.0004, q=0.0004; aripiprazole versus aripiprazole+α-lipoic acid, χ^2^=13.81, *P*=0.0002, q=0.0004; control versus aripiprazole+α-lipoic acid, χ^2^=0.0103, *P*=0.9193, q=0.3371). (D,E) Expression of Sod2 reduced the number of cells positive for the active caspase Dcp-1 in *Drosophila* kept on an aripiprazole-supplemented diet. (D) Representative images showing active Dcp-1 (green) and cell nuclei (magenta). (E) Quantification of active Dcp-1 in the posterior midgut. Bars show mean±s.e.m. ****P≤0.0001 (one-way ANOVA with Holm-Šídák's multiple comparisons test). (F) Defects in the intestinal barrier observed in aripiprazole-supplemented flies were reduced by expression of Sod2. **P≤0.01 (χ^2^ two-sided test with analysis of a stack of *P*-values, FDR=10%; control+normal diet versus control+aripiprazole, χ^2^=7.457, *P*=0.0063, q=0.0063; control+aripiprazole versus *mex>Sod2*+aripiprazole, χ^2^=8.457, *P*=0.0036, q=0.0036; control+normal diet versus *mex>Sod2*+aripiprazole, χ^2^=0.002257, *P*=0.9621, q=0.9621). (G,H) Expression of the yeast alternative oxidase *NDI1* reduced the number of cells positive for the active caspase Dcp-1 in *Drosophila* kept on an aripiprazole-supplemented diet. (G) Representative images showing active Dcp-1 (green) and cell nuclei (magenta). (H) Quantification of active Dcp-1 in the posterior midgut. Bars show mean±s.e.m. *P≤0.05 (one-way ANOVA with Holm-Šídák's multiple comparisons test). Genotypes: *w^1118^CS* (A-C); *w; mex1-Gal4/UAS3M; +* (control) and *w; mex1-Gal4/UAS-Sod2* (*mex>Sod2*) (D-F); and *w; mex1-Gal4/UAS3M; +* (control) and *w; mex1-Gal4; UAS-NDI1* (*mex>NDI1*) (G-H).

We next tested the consequences of reducing ROS levels on intestinal integrity and cell death in the digestive tract of flies kept on an aripiprazole-supplemented diet. We found that in flies kept on a diet supplemented with both aripiprazole and α-lipoic acid, there was a reduction in the number of cells positive for the active effector caspase Dcp-1 compared to flies kept on a diet supplemented with aripiprazole alone (Fig. 7A,B). We next examined the gut barrier integrity of flies kept on these different diets. We found that combining aripiprazole and α-lipoic acid in the diet reduced the defects in gut barrier integrity (Fig. 7C). Next, we overexpressed mitochondrial superoxide dismutase 2 (Sod2) in the enterocytes of aripiprazole-supplemented flies. Sod2 catalyses the dismutation of superoxide anions into hydrogen peroxide and molecular oxygen. This reaction is critical for detoxifying superoxide radicals generated as byproducts of mitochondrial respiration (reviewed in Dosunmu-Ogunbi et al., 2019). Flies overexpressing Sod2 and kept on a diet containing aripiprazole had less intestinal cell death (Fig. 7D,E) and an improved gut barrier integrity (Fig. 7F) compared to control flies kept on the same diet.

The expression of *NDI1* reduced the levels of ROS in the intestines of flies kept on a diet supplemented with aripiprazole (Fig. 5). We next tested whether its expression could also decrease the degree of caspase activation in the intestine of flies kept on an aripiprazole-containing diet. Expressing *NDI1* using an enterocyte driver in flies kept on an aripiprazole-supplemented diet decreased in the number of cells positive for Dcp-1 (Fig. 7G,H). We conclude that the off-target toxicity of aripiprazole in the intestine of flies involves mitochondrial ROS that trigger JNK-dependent apoptosis in enterocytes.

### Intestinal cell proliferation is not altered by aripiprazole

**Fig. 8.**
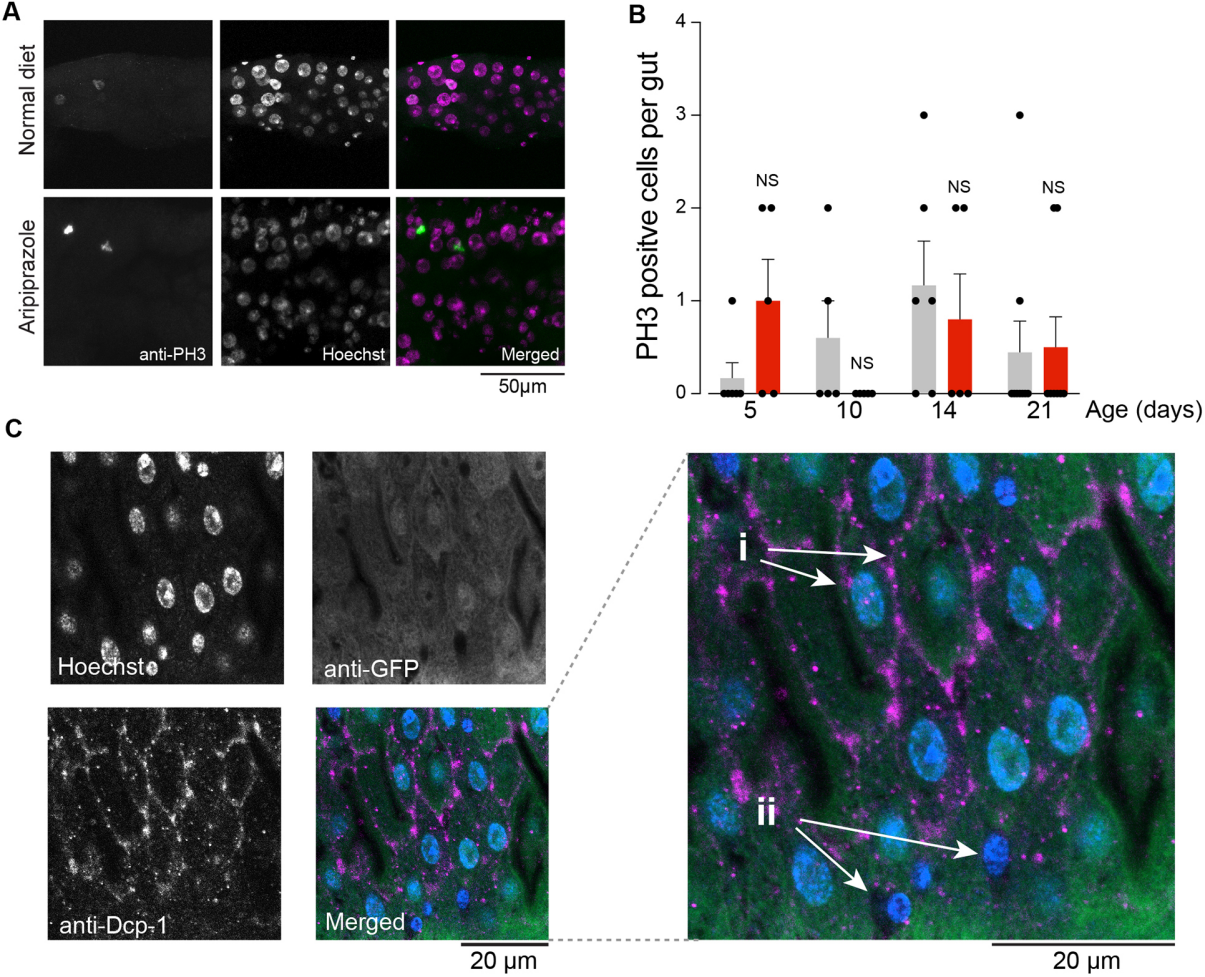
**Aripiprazole supplementation does not alter cellular proliferation and promotes enterocyte apoptosis.** (A,B) An aripiprazole-supplemented diet did not alter the number of proliferating cells in the gut. Proliferation was assessed by counting cells positive for phospho-histone H3 (PH3), a proliferation marker. (A) Representative images of adult fly gut showing PH3 staining (green) and cell nuclei (magenta). (B) Quantification of PH3-positive cells in the entire midgut. Bars show mean±s.e.m. NS, not significant; *P*≥0.05 (Holm-Šídák's multiple comparisons test). (C) An aripiprazole-supplemented diet results in enterocyte death. Representative images of a section of the adult fly intestine expressing GFP using an enterocyte-specific marker, *mex1*, stained for GFP (green), active Dcp-1 (magenta) and cell nuclei (blue). In the magnified view on the right, GFP-positive enterocytes show positive staining for active Dcp-1 (i), and two other cells negative for the *mex1*-driven GFP marker and also negative for active Dcp-1 (ii) are indicated by white arrows. Images are representative of at least five guts. Genotypes: *w^1118^CS* (A,B); *w; mex1-Gal4/UAS-GFP; +* (C)*.*

Intestinal cell death results in a proliferative response by intestinal stem cells in flies (Nászai et al., 2015; Popovic et al., 2023). Because flies kept on an aripiprazole-containing diet showed a significant increase in intestinal apoptosis, we next measured the degree of cellular proliferation in flies kept on an aripiprazole-supplemented diet by monitoring the number of phospho-histone H3 (PH3)-positive cells. Histone H3 is a component of the nuclear chromatin and is phosphorylated during mitotic cell division. We found no significant changes in the number of PH3-positive cells in flies supplemented with aripiprazole across the period of exposure to the drug (Fig. 8A,B). Finally, we confirmed that the cell death observed in the intestine of flies kept on an aripiprazole-supplemented diet was occurring in the enterocytes. We expressed GFP using *mex1*, an enterocyte driver, and monitored the levels of active Dcp-1 when these flies were kept on an aripiprazole-supplemented diet (Fig. 8C). We found that GFP expression colocalized with active caspase staining. We conclude that the cell death observed in flies kept on an aripiprazole-supplemented diet occurs mostly in intestinal enterocytes without triggering a proliferative response by intestinal stem cells.

## DISCUSSION

Here, we observed that oral intake of aripiprazole caused mitochondrial dysfunction, increased levels of ROS, activated the JNK pathway in the digestive system and increased caspase activation. Our observations in *Drosophila* build on our previous finding that aripiprazole causes mitochondrial dysfunction (Hardy et al., 2023). This evidence is also consistent with a report that the oral administration of aripiprazole to mice increased the levels of phospho-c-Jun and induced the processing of caspase-3, an executioner caspase (reviewed in Earnshaw et al., 1999), in the intestine (Baek et al., 2015). Taken together, these observations and our present data indicate that aripiprazole induces intestinal toxicity by activating JNK-dependent apoptotic cell death.

The JNK pathway can be activated by the extrinsic tumour necrosis factor (TNF) superfamily ligand Egr (also known as Eiger or TNFα), which engages two TNF receptors, or by intrinsic factors, such as apoptosis signal-regulating kinase 1 (dASK1) (reviewed in Herrera and Bach, 2021). dASK1 is a serine threonine kinase that is activated by several different stressors, including genotoxic stress, endoplasmic reticulum stress, serum or trophic factor withdrawal and ROS. dASK1 activates the JNK pathway, which leads to apoptosis (reviewed in Shiizaki et al., 2013). Pro-apoptotic proteins, such as Reaper, also activate JNK signalling via the degradation of inhibitors of apoptosis proteins, such as DIAP1, and the subsequent stabilisation of TNF receptor-associated factor (dTRAF1) and dASK1 (Kuranaga et al., 2002). Ultimately, the fate of cells in which JNK is activated depends on the duration of exposure and intensity of the stressor. The flies in our experiments were continuously exposed to aripiprazole for 21 days, and at the end of this period, most enterocytes in the gut were positive for the JNK reporter and active caspase Dcp-1. Therefore, the prolonged mitochondrial stress and ROS generated in gut enterocytes resulted in sustained JNK activation and cell death.

In *Drosophila*, the death of enterocytes induces epithelial renewal by increasing the proliferation of intestinal stem cells (Buchon et al., 2009). Aripiprazole induces apoptosis in enterocytes, but we observed a lack of compensatory epithelial renewal. Mitochondria play an important role in regulating the proliferation of intestinal stem cells (reviewed in Morris and Jasper, 2021). Excessive ROS, or the inhibition of oxidative phosphorylation complexes, can block the division of intestinal stem cells and the subsequent differentiation of enterocytes (Zhang et al., 2020). A loss of compensatory proliferation due to high levels of ROS, inhibition of oxidative phosphorylation, or cell death of intestinal stem cells may compound toxicity and further contribute to intestinal dysfunction.

Loss of intestinal integrity in *Drosophila* correlates with a reduced lifespan. Previous studies showed that at 20 days, Smurf-positive flies had a significantly shorter lifespan than Smurf-negative flies (Rera et al., 2011). Although we observed a large increase in the number of flies with a loss of intestinal integrity, there was no difference in the lifespan of the flies kept on an aripiprazole-supplemented diet. The activation of JNK protects against ageing and extends lifespan (Wang et al., 2003). We hypothesise that the sustained activation of JNK signalling might sustain the lifespan of flies supplemented with aripiprazole.

The observed intestinal defecation side effects may also result from the on-target effects of aripiprazole on neurotransmitter receptors. *Drosophila* express dopamine receptors in the midgut (El Kholy et al., 2021). However, these are predominantly the Dop1R1 receptors, which are the closest orthologues of the human D1 receptors. The expression of the orthologue of the human D2 receptor (Dop2R), to which aripiprazole binds with high affinity (Shapiro et al., 2003), was not detected in studies of the adult *Drosophila* midgut (Hung et al., 2020). Dietary supplementation with dopamine in flies increased faecal output and suppressed the expression of dopamine receptors, which decreased peristalsis (El Kholy et al., 2021). This effect on faecal output is likely not direct because the neural control of defecation in *Drosophila* predominantly involves glutaminergic neurons (Zhang et al., 2014).

In contrast to *Drosophila*, increased dopaminergic signalling in mammals exerts a mostly inhibitory effect on gut function (reviewed in Serio and Zizzo, 2023). Aripiprazole is a partial agonist of D2 receptors that acts to stabilise dopaminergic signalling. Full D2 receptor antagonists, such as domperidone and metoclopramide, are licenced for use as antiemetics and prokinetics in the upper gastrointestinal tract (Puoti et al., 2023). In mammals, dopamine receptors are expressed in the pancreas, intestinal mucosa and smooth muscle (reviewed in Zhang et al., 2021). Although the relationship between modulation of dopaminergic signalling and changes in ROS levels, JNK activation and cell death is established in neuronal cells (Luo et al., 1998), it is yet to be determined whether *Drosophila* intestinal cells exhibit a similar pathway. As the D2R receptor is not found in enterocytes (Hung et al., 2020), we can conclude that the mitochondrial dysfunction caused by aripiprazole in the gut is responsible for the death of intestinal cells and the impairment of intestinal function.

Our study suggests that the co-administration of aripiprazole and an antioxidant mitigates any off-target gut toxicity. There is some evidence for the use of antioxidants to reduce the side effects of pharmaceuticals (reviewed in Forman and Zhang, 2021). Antioxidants have been co-administered with chemotherapy drugs to reduce side effects (Yasueda et al., 2016) and are commonly used in the treatment of paracetamol-induced liver toxicity (Chiew et al., 2020; Tenório et al., 2021). However, the unregulated use of dietary supplements, including antioxidants, presents a potential risk to patients because of the possibility of reducing drug efficacy or causing additional side effects. The co-administration of sinapic acid, an antioxidant, and aripiprazole in rats results in a significantly greater plasma concentration of aripiprazole than the administration of aripiprazole alone because of the inhibition of CYP450 enzymes, which may increase the risk of overdose (Raish et al., 2019). These results suggest that the choice and dose of a specific antioxidant should be considered when combining antioxidants with pharmacological drugs.

## MATERIALS AND METHODS

### Analysis of the side effects of aripiprazole in humans

We queried the FDA Adverse Event Reporting System (FAERS) database (https://open.fda.gov/data/faers/, accessed in May 2024) for the side effects of oral aripiprazole using the Public Dashboard. This database contains information provided by patients or their health care providers. This information is collated by the MedWatch program of the FDA for reporting serious reactions, product quality problems, therapeutic inequivalence or failure, and product use errors with human medical products, including drugs, biological products, medical devices, dietary supplements, infant formula and cosmetics. The data were arbitrarily filtered to include only patients with a recorded age greater than 5 years to avoid records pertaining to prenatal exposure. We obtained descriptive statistics on the side effects of aripiprazole. Some reports contained multiple side effects. Therefore, to calculate the percentage of patients experiencing a gut side effect, we searched the list of reactions and compared it against the list of gastrointestinal side effects used in the FAERS database. We assigned reaction reports a true or false status for gastrointestinal problems to calculate the number of reports that included at least one reaction. To calculate the total number of reactions for each specific gastrointestinal side effect, we divided each report into single reactions and plotted the top ten reported gastrointestinal side effects.

### *Drosophila* strains

Fly stocks were maintained on standard cornmeal agar media at 25°C. The strains used were: *w^1118^CS* (*Canton-S* backcrossed to *w^1118^*), *puc^E69^/TM3, Sb^1^* (98392), *w; P{mex1-Gal4}* (91368), *UAS-Sod2* (24494) and *UAS-Puc* (98328) from the Bloomington *Drosophila* Stock Center; *UAS-GFP* (Fly Facility, Department of Genetics, University of Cambridge); *UAS-3M* empty vector (a kind gift from Flaviano Giorgini, Department of Genetics, University of Leicester); and *UAS-NDI1* (a kind gift from Alex Whitworth, MRC Mitochondrial Biology Unit, University of Cambridge).

### Drug treatments

Aripiprazole (Acros Organics, 15452192) dissolved in DMSO (100 mM) was added to the fly food at a final concentration of 1 mM with 1% DMSO. Flies kept on a diet supplemented with aripiprazole were compared to control flies kept on a diet supplemented with 1% DMSO. For experiments involving antioxidants, α-lipoic acid (Sigma-Aldrich, 437692) was dissolved in DMSO (215 mM). Diets containing aripiprazole and α-lipoic acid were prepared by combining both chemicals in liquid food at final concentrations of 1 mM and 2.15 mM, respectively. The concentration of α-lipoic acid used was described by Lang et al. (2021). The concentration of aripiprazole in the flies was measured after 14 or 21 days of the administration of drug-supplemented food. Flies were snap frozen in liquid nitrogen and stored at −80°C until drug extraction. Five flies per condition per replicate were homogenised using a motorised pestle in 1.5-ml microcentrifuge tubes in 250 µl of ice-cold extraction solvent (40% methanol, 40% acetonitrile and 20% water by volume, containing 20 µM caffeine as an internal standard) and frozen at −80°C. For calibration of the assay, flies kept on normal food were homogenised in different standard concentrations (from 0.001 to 20 µM) of aripiprazole before freezing at −80°C. The frozen samples at −80°C were thawed on ice, and two further cycles of homogenisation, freezing at −80°C and thawing were performed. The lysates were cleared via centrifugation (20,000 ***g*** for 10 min at 4°C) and the cleared supernatants were stored at −20°C. The pellets were resuspended in 250 µl of fresh ice-cold extraction solvent and the previous homogenisation/freeze/thaw cycle was performed three times. The supernatants from the two cycles of extractions were subsequently combined and dried using a SpeedVac vacuum concentrator and stored at −80°C until analysis. For analysis, the dried samples were dissolved in 100 µl of reconstitution solvent (80% v/v methanol/water) and analysed using liquid chromatography (LC) coupled with mass spectrometry (MS).

LC-MS analysis was performed on an Agilent 1290 Infinity II LC system coupled with an Agilent 6546 LC/quadrupole time-of-flight (Q-TOF) mass spectrometer. The Q-TOF MS scan was operated in positive mode using four different collision energies (0, 10, 20 and 40 V) (30-1500 m/z). The source parameters were as follows: gas temperature, 200°C; drying gas, 9 l/min; nebulizer, 1.38 bar; sheath gas temperature, 400°C; sheath gas flow, 12 l/min; capillary voltage, 3000 V; nozzle voltage, 0 V, fragmentor, 110 V; skimmer, 45 V; Oct RF Vpp (peak-to-peak voltage applied to an octopole ion guide), 750 V. The online mass calibration was performed using a reference solution (121.05 and 922.01 m/z). The separation was performed using a ZORBAX RRHD Eclipse Plus column (C18, 2.1×100 mm, 1.8 μm; Agilent, 858700-902) with a ZOBRAX Eclipse Plus guard column (C18, 2.1×5 mm, 1.8 μm; Agilent, 821725-901) at 40°C. The multisampler was kept at a temperature of 4°C and the injection volume was 1 μl. The flow rate was 0.4 ml/min. The mobile phases comprised solvents A (water+0.1% formic acid+5 mM ammonium formate) and B (methanol+0.1% formic acid+5 mM ammonium formate). The 15 min gradient started with 5% solvent B, which was increased to 30% over 1 min and then further increased to 100% by 7 min and held for 3 min, before returning to 5% solvent B for a 5 min re-equilibration. Pure standards were used for compound identification and calibration, and compound identification was based on retention time, accurate mass and fragmentation patterns. The Agilent MassHunter Qualitative Analysis 10.0 software was used to qualify the selected compound standards. Total ion chromatogram, extracted ion chromatogram (EIC) and EIC fragment graphs were extracted for each compound. The Agilent MassHunter TOF Quantitative Analysis (version 10.1) software was used to quantify compounds in each sample.

A calibration curve was obtained from the ratio of known concentrations of aripiprazole/caffeine. The ratio obtained from the aripiprazole-supplemented flies was used to determine the concentration of aripiprazole in the samples from the standard curve. From this, the mass of aripiprazole present in the sample was calculated and divided by 5 to give an average mass per fly. The volume of a fly was calculated by assuming a fly is a cuboid bounded by three dimensions: proboscis to anal plate, tip of thorax to leg base, and distance between eyes. Four male wild-type (*w^1118^Cs w+*) flies were examined, giving an average volume of 1.149 mm^3^. Using the average mass of aripiprazole per fly and the average fly volume, the concentration of aripiprazole was calculated.

### Lifespan analysis

Groups of 15 newly eclosed males of each genotype were placed into separate vials containing food supplemented with aripiprazole or 1% DMSO. The flies were transferred to vials containing fresh food every 3 to 4 days, and the number of dead flies was recorded. The data are presented as Kaplan‒Meier survival curves, and significance was determined by log-rank tests.

### Analysis of faecal deposits

21-day-old male flies were sorted into groups of five and placed in small dishes containing a small 2×2 mm piece of food supplemented with 2.5% (w/v) erioglaucine disodium salt (Sigma-Aldrich, 861146) to produce a blue colour for 16 h overnight. The next day, the flies and food were removed, and the dishes were scanned with a flatbed scanner (Epson Perfection, V750 Pro). Images were cropped and analysed using TURD software (Wayland et al., 2014).

### Capillary feeding assay

Flies were starved in an empty plastic fly vial with cotton wool soaked in water for hydration for 3 h prior to the start of the assay. The flies were sorted into groups of five and placed in tubes with three microcapillary pipettes (VWR, 612-1401), each containing 5 µl of liquid food (Ja et al., 2007). Liquid food was prepared by adding 50 mg of yeast to 1.5 ml of water and heating to 99°C. The resulting mixture was centrifuged, and 43 mg of sucrose was added to 800 µl of the supernatant with 1.5 µl of erioglaucine disodium salt to visualise the air–liquid interface. Food consumption was measured 18 h later using an electronic calliper (Dasqua Bluetooth Digital Calliper 12″/300 mm, 24108120).

### Assay of intestinal barrier integrity (Smurf assay)

We measured the integrity/functionality of the intestinal barrier by detecting the presence of a non-absorbable, non-toxic blue food dye outside the digestive tract after feeding (Rera et al., 2011). The flies were transferred to food supplemented with 2.5% (w/v) erioglaucine disodium salt and left overnight (Martins et al., 2018). The following morning, the presence of dye outside the upper abdomen was measured and scored. We did not score different degrees of ‘Smurfness’. A pairwise test, followed by a stack of *P*-values with a false discovery rate (FDR) of 10%, was used to detect the significance of differences between genotypes.

### Assessment of ATP levels

We measured the ATP levels of flies as described (Costa et al., 2013). Briefly, we collected five flies per replicate in 1.5 ml tubes, which were fed aripiprazole-supplemented food for 14 days. The flies were homogenised using motorised pestles in 100 µl of homogenisation buffer (100 mM Tris and 4 mM EDTA, pH adjusted to 7.8). Samples were frozen in liquid nitrogen, followed by heating to 95°C for 5 min. Samples were centrifuged at 600 ***g*** for 10 min and diluted (1:50) in the homogenisation buffer. 100 µl of the diluted sample was added to a white solid-bottom plate with 100 µl of the CellTiter-Glo solution (CellTiter-Glo Luminescent Cell Viability Assay, Promega, Fitchburg, WI, USA) to each well. Samples were mixed on an orbital shaker while protected from light for 5 min and luminescence was measured immediately on an Infinite M200Pro multifunction reader (TECAN, Männedorf, Switzerland). Luminescence values were normalised to protein concentration calculated by using the bicinchoninic acid assay.

### Microscopy-based assessment of mitochondrial function and ROS levels

The Δψm in *Drosophila* midguts was measured as previously described (Popovic et al., 2023). Briefly, midguts were loaded with 40 nM TMRM (Thermo Fisher Scientific, T668) in loading buffer (10 mM HEPES pH 7.35, 156 mM NaCl, 3 mM KCl, 2 mM MgSO_4_, 1.25 mM KH_2_PO_4_, 2 mM CaCl_2_ and 10 mM glucose) for 30 min at room temperature, and the dye was present during the experiment. In these experiments, TMRM was used in redistribution mode to assess the Δψm. Therefore, a reduction in TMRM fluorescence represents mitochondrial depolarisation. Confocal images were obtained using a Zeiss LSM 880 confocal microscope equipped with a 20× air objective. The illumination intensity was maintained at a minimum (at 0.1-0.2% of the laser output) to avoid phototoxicity, and the pinhole was set to acquire an optical slice of 2 μm. The fluorescence was quantified by exciting TMRM using a 565 nm laser and measured above 580 nm. *Z*-stacks of five 300-μm^2^ fields per brain were acquired, and the mean maximal fluorescence intensity was measured for each group.

To measure ROS, the guts of male flies were dissected in ice-cold PBS and incubated with 5 μM MitoSOX Red mitochondrial superoxide indicator (Molecular Probes, M36008) for 20 min. After incubation, the samples were washed with PBS for 10 min and immediately imaged on a Zeiss LSM 880 confocal microscope. The maximal-intensity projections of the MitoSOX signal in the fly gut were quantified using ImageJ.

### Immunofluorescence and confocal microscopy

For imaging, intestines were dissected from live flies in ice-cold PBS, fixed in 4% paraformaldehyde for 30 min and blocked overnight in blocking buffer (10% normal goat serum in PBS containing 0.5% Triton X-100). The samples were then incubated with primary antibodies [anti-cleaved *Drosophila* Dcp-1 (Cell Signaling Technology, 9678, 1:100), anti-GFP (Abcam, ab13970, 1:1000) and anti-β-galactosidase (Promega, Z378A, 1:500)] at 4°C overnight, followed by incubation with secondary antibodies [Alexa Fluor 488-conjugated F(ab′)2 fragment of goat anti-rabbit IgG (H+L) (Invitrogen, A11070, 1:200), Alexa Fluor 488-conjugated goat anti-chicken IgY (H+L) (Abcam, ab150169, 1:200), ANTI Rb Far RED or Alexa Fluor 488-conjugated goat anti-mouse IgG (H+L) (Invitrogen, A11029, 1:200)] and Hoechst 33342 (Invitrogen, H3570, 1:500) for 30 min at room temperature. Staining of cells positive for PH3 was performed by incubating the intestines with a mixture of 1:1 anti-phospho-histone H3 (Ser10) antibody (Cell Signaling Technology, 9701) and anti-phospho-histone H3 (Ser28) antibody (Cell Signaling Technology, 9713) diluted 1:200 in blocking buffer. Confocal images were acquired using a Zeiss LSM 880 or 980 system.

### Digital image processing

Fluorescence images and metadata were acquired in the ZVI format and exported as uncompressed bitmapped digital data (TIFF format). They were processed using ImageJ with established scientific imaging workflows.

### Statistical analyses

Statistical analyses were performed using GraphPad Prism (https://www.graphpad.com/). The data are presented as the mean values, and the error bars indicate the s.e.m. The number of biological replicates per experimental variable (*n*) is indicated in the respective figure or figure legend. No sample was excluded from the analysis unless otherwise stated. Samples were not anonymized. For all the statistical analyses, the D'Agostino and Pearson tests were used to determine whether the data followed a normal distribution. Based on the normality test results, parametric or nonparametric analysis was used. Significance is indicated as: NS, not significant; *P*≥0.05; *P≤0.05; **P≤0.01; ***P≤0.001; and ****P≤0.0001.
